# A case of reversible cerebral vasoconstriction syndrome developing during treatment of adult aplastic anemia

**DOI:** 10.1007/s00277-018-3430-6

**Published:** 2018-07-10

**Authors:** Akira Yamamoto, Yusuke Meguri, Akiko Fukuda, Yui Kambara, Tomohiro Urata, Taiga Kuroi, Taro Masunari, Nobuo Sezaki, Toru Kiguchi

**Affiliations:** 1 Department of Hematology, Chugoku Central Hospital of the Mutual Aid Association of Public School Teachers, 148-13, Kamiiwanari, Miyuki-Cho, Fukuyama, 720-0001 Japan; 2 Department of Hematology, Oncology and Respiratory Medicine, Okayama University Graduate School of Medicine, Dentistry and Pharmaceutical Sciences, Okayama, Japan; 3 Center for Graduate Medical Education, Okayama University Hospital, Okayama, Japan

Dear Editor,

Reversible cerebral vasoconstriction syndrome (RCVS) refers to a group of disorders characterized by “thunderclap headaches” and diffuse narrowing of the cerebral arteries [1]. RCVS sometimes develops after the use of immunosuppressants that are frequently used to treat hematological diseases. However, only two pediatric cases of RCVS in patients with a hematological disease have been reported [2, 3]. Herein, we report the first adult case of RCVS in a patient with aplastic anemia (AA).

**Fig. 1:**
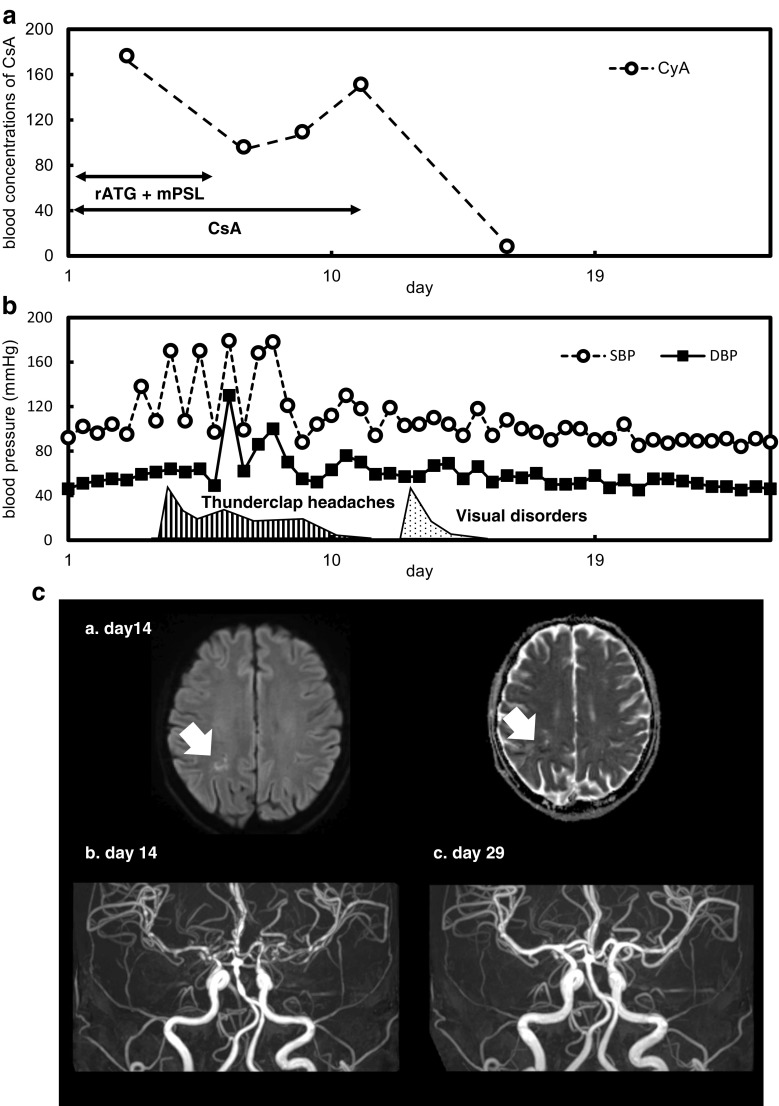
The progress of treatment, with symptoms and neuroimages. **a** The blood concentrations of CsA (ng/mL) and the times of prescription to treat severe AA. **b** Blood pressure (circles: SBP; squares: DBP; mmHg) and symptoms over time (stripes: thunderclap headaches from day 5 to 11; polka-dots: visual disorders from day 13 to 15). **c** (a.) The MRI DWI map (left) and the MRI ADC map (right) reveal fresh cerebral infarction of the left occipital lobe (arrows) on day 14 after treatment commenced. (b.) MRA of the arterial circle of Willis reveals segmental vasoconstrictions of the basilar artery, the anterior and posterior communicating arteries, and the anterior and middle cerebral arteries, on day 14 after treatment commenced. (c.) MRA of the arterial circle of Willis reveals diffuse improvement of vasoconstriction on day 29 after treatment commenced. CsA cyclosporine A, AA aplastic anemia, ATG anti-thymocyte globulin, mPSL methylprednisolone, SBP systolic blood pressure, DBP diastolic blood pressure, MRI magnetic resonance imaging, DWI diffusion-weighted imaging, MRA magnetic resonance angiography, ADC apparent diffusion coefficient

A 58-year-old female presented with a severe headache and hypertension on day 5 after commencement of oral cyclosporine A (CsA) and rabbit anti-thymocyte globulin (ATG) to treat severe AA. Initially, she was given analgesics for the headache and CsA because head CT scans showed no evidence of bleeding of the brain. However, her headache persisted, and we stopped CsA on day 11. Thereafter, her headache ceased, but on day 13, she complained of a bilateral visual field defect. Magnetic resonance imaging revealed multiple small cerebral infarctions, and magnetic resonance angiography (MRA) revealed diffuse vasoconstrictions of the cerebral arteries (Fig. 1). Her neurological findings and cerebral images gradually improved. On day 217, she was retreated with low-dose CsA and exhibited no neural sequelae. The blood cell numbers increased, but she remained transfusion-dependent (except for platelets); we scheduled monthly erythrocyte transfusions.

It is important to reduce or stop a drug that is causing RCVS, such as CsA and rabbit-ATG [2, 3]. The hypertension and thunderclap headaches ceased immediately after CsA was stopped, but the possibility that rabbit-ATG induced RCVS cannot be discounted.

Magnesium sulfate [4] and calcium antagonists [3, 5] are useful RCVS therapies, reducing blood pressure and dilating the cerebral vessels. Retreatment of AA with CsA is sometimes inevitable; Ueki et al. retreated AA by CsA with lomerizine without relapsing RCVS [3]. As the hypertension improved after CsA was stopped, we did not use calcium antagonists.

We commenced CsA at a low dose and controlled the blood level because side-effect development depends on the CsA blood concentration [6]. A switch to tacrolimus is one possible strategy when posterior reversible encephalopathy syndrome (PRES) develops [7]. Tacrolimus also causes RCVS [8], but is effective against AA [9]. The class effect of the various calcineurin inhibitors on RCVS remains poorly known; these materials may be useful treatment options.

A diagnosis of RCVS requires MRA, but MRA findings are sometimes not initially apparent; repeat MRA is recommended ≥ 1 week after onset [10]. Only two cases of RCVS in patients with hematological diseases have been reported despite many such patients taking drugs that can induce RCVS. The condition may be misdiagnosed, because MRA is not routine; possible erroneous diagnoses include PRES. The prognosis of RCVS is thought to be good, but severe complications such as cerebral hemorrhage and infarctions can develop. Accurate diagnosis via MRA is essential; more cases are required to study whether to restart CsA or switch to tacrolimus to treat AA with a history of RCVS.
